# Clinical Characteristics of Patients with Long-Bone Fracture Nonunion and Delayed Union and Factors Associated with Infection: A Retrospective Single-Center Cohort Study

**DOI:** 10.3390/jcm15135008

**Published:** 2026-06-27

**Authors:** Dina Saginova, Marina Sorokina, Airat Syundyukov, Assel Kaliyeva, Yersultan Alzhanov, Arsen Kaliyev

**Affiliations:** 1 National Scientific Center of Traumatology and Orthopedics Named After Academician N.D. Batpenov, 15a Abylai Khan Street, Astana 010000, Kazakhstan; saginova_d@nscto.kz (D.S.); asselkyz@gmail.com (A.K.); ersyltan@gmail.com (Y.A.); 2 Karaganda Medical University, 40 Gogol Street, Karaganda 100000, Kazakhstan; m.sorokina@qmu.kz; 3 Department of Traumatology, Orthopedics and Emergency Medicine, I.N. Ulyanov Chuvash State University, Cheboksary 428003, Russia; sndk-ar@yandex.ru

**Keywords:** fracture nonunion, delayed union, pseudarthrosis, osteomyelitis, revision osteosynthesis

## Abstract

**Background/Objectives:** To evaluate the clinical and demographic characteristics of patients with impaired union of long bone fractures admitted to a specialized orthopedic center and to identify factors associated with infection on admission. **Methods:** A retrospective, single-center cohort study was conducted at the National Scientific Center of Traumatology and Orthopedics named after Academician N.D. Batpenov. The study included patients hospitalized between 2023 and 2025 with diagnoses of fracture nonunion or delayed union. Demographic characteristics, lesion location, interval from injury to hospitalization, previous treatment, presence of revision osteosynthesis, infection on admission, and length of hospitalization were analyzed. Univariate analysis and multivariate logistic regression were used to identify factors associated with infection. Patients with osteomyelitis were excluded from the regression model to avoid definitional collinearity. **Results:** During the study period, 360 hospitalizations were recorded in 336 unique patients. The annual incidence increased from 79 in 2023 to 166 in 2025. The median patient age was 50 years, with women accounting for 52.5% of the sample. The most common bone sites were the femur (36.1%), humerus (23.6%), and tibia (15.0%). The median interval from injury to hospitalization at a specialized center was 2 years. Prior revision osteosynthesis was noted in 34.2% of patients. Infection on admission was detected in 20.3% of patients and was associated with a longer hospital stay. In an exploratory multivariable model (EPV ≈ 2.8), previous revision osteosynthesis was associated with infection on admission (OR 8.26; 95% CI 2.76–24.74; *p* < 0.001). **Conclusions:** Patients with nonunion and delayed union of long-bone fractures referred to a specialized center represent a clinically complex population characterized by prolonged time from injury, previous surgical interventions, and a substantial burden of infection. In an exploratory multivariable analysis, previous revision osteosynthesis was associated with infection on admission and may represent a marker of clinical complexity and prior treatment burden rather than a causal determinant of infection. Further prospective studies are required to clarify factors associated with infection and treatment outcomes in this patient population.

## 1. Introduction

Fracture nonunion, including delayed union and nonunion, can result from patient-related factors such as smoking, obesity, alcohol consumption, and comorbidities, as well as injury-related factors including open or multiple fractures, compromised blood supply, infection, and inadequate stabilization [1].

The clinical and economic consequences of fracture nonunion are significant: impaired limb function, chronic pain, the need for repeated interventions, long-term disability, and decreased quality of life [2]. Furthermore, nonunion can lead to chronic deformity, instability, and the progression of degenerative joint disease requiring complex surgical treatment [1].

Several factors contribute to impaired reparative osteogenesis, including fracture location, the extent of soft tissue injury, infection, and systemic diseases such as advanced age, metabolic disorders, and diabetes mellitus [3]. In recent years, particular attention has been paid to obesity and body mass index as potentially modifiable risk factors affecting fracture healing and postoperative complications [3,4].

However, most large-scale registry-based studies lack detailed clinical variables, such as comorbidity burden, local biomechanical conditions, and treatment-specific factors, which are essential for understanding the real-world outcomes of patients with impaired fracture healing. Therefore, single-center studies that comprehensively assess patient characteristics and clinical factors remain crucial, particularly in healthcare systems undergoing transformation.

Despite the availability of large international studies, data on the clinical profile of patients with impaired fracture healing referred to specialized centers in Central Asian countries remain limited. In the context of interregional routing, such cohorts may differ significantly from the general fracture patient population, as they concentrate more severe, long-standing, and previously operated cases. This highlights the need to analyze local clinical data to assess patient composition, prior treatment burden, and factors associated with infection.

The aim of this study was to evaluate the clinical and demographic characteristics of patients with fracture nonunion and delayed union treated at a specialized center and to identify factors associated with infection at admission.

## 2. Materials and Methods

### 2.1. Study Design

This study was a retrospective, single-center cohort study. It was conducted at the National Scientific Center of Traumatology and Orthopedics named after Academician N.D. Batpenov (NSCTO), a leading specialized center in the Republic of Kazakhstan in the field of traumatology and orthopedics. The center receives patients with complex bone and joint pathologies from all regions of the country, which determines the interregional nature of the study cohort.

The analysis included patients hospitalized between January 2023 and December 2025 with diagnoses consistent with impaired long bone fracture consolidation.

The study was observational and aimed to evaluate the clinical and demographic characteristics of the patients, as well as factors associated with the development of infectious complications. 

### 2.2. Inclusion and Exclusion Criteria

The study included patients aged 16 years or older who were hospitalized at NSCTO during the specified period with a diagnosis of long bone fracture nonunion (M84.1) or delayed union (M84.2). Nonunion is generally diagnosed when there is no radiographic evidence of progression toward healing over at least 3 consecutive months, and the fracture is considered unlikely to unite without further intervention, typically ≥9 months after injury. Delayed union is considered when fracture healing progresses more slowly than expected for the specific fracture type and time since injury, without fulfilling criteria for established nonunion.

Cases with incomplete clinical data, tumor bone lesions, and nontraumatic pathological fractures were excluded. Patients with chronic osteomyelitis (M86.x) were included in the descriptive analysis but were not included in the multivariable model evaluating factors associated with infection due to the definitional dependence of the outcome.

### 2.3. Data Collection and Study Variables

Data were extracted from inpatient medical records. Age, gender, body mass index, fracture location, type of nonunion (M84.1/M84.2), open nature of the primary fracture, interval from injury to hospitalization at NSCTO, presence of diabetes mellitus and other registered comorbidities, method of primary treatment, previous revision osteosynthesis, type of surgery at NSCTO, and use of autologous bone grafting were analyzed. The primary outcome was infection at admission.

Infection was defined as the presence of clinical signs of infection at the nonunion site, including fistula, purulent discharge, inflammatory changes in soft tissues, intraoperative signs of infection, or a diagnosis of chronic osteomyelitis. Cases with M86.4/M86.6 were classified as infectious. Bacterial culture data and laboratory markers of inflammation were considered when available but were not used as mandatory criteria due to the retrospective design. In cases of discrepancy between clinical signs and the diagnostic code, priority was given to the clinical data: the presence of a fistula, purulent discharge, or intraoperative signs of infection were sufficient criteria, regardless of the diagnostic code. The length of hospitalization was additionally assessed as an indicator of the severity of the disease. Infection status was adjudicated during the index hospitalization based on all available clinical, intraoperative, coding, microbiological, and laboratory information.

### 2.4. Statistical Analysis

Statistical analysis was performed using IBM SPSS Statistics for Windows, Version 27.0 (IBM Corp., Armonk, NY, USA) and GraphPad Prism (GraphPad Software, San Diego, CA, USA). The normal distribution of quantitative variables was assessed using the Shapiro–Wilk test. Quantitative data are presented as mean ± standard deviation for a normal distribution or median and interquartile range for a non-normal distribution; categorical variables are presented as absolute values and percentages. Between-group comparisons were performed using the Mann–Whitney test, Pearson’s χ^2^ test, or Fisher’s exact test for expected frequencies <5. A *p*-value < 0.05 was considered statistically significant. Factors associated with infection on admission were assessed first in univariate analysis and then in multivariate logistic regression, including clinically significant variables: age, gender, body mass index, diabetes mellitus, interval from injury to hospitalization at NSCTO, and previous revision osteosynthesis. Patients with osteomyelitis (M86.x) were excluded from the regression model to avoid definitional collinearity, since infection is a diagnostic component of this nosology. Open fractures were not included in the multivariate model due to complete separation of the data: infection was observed in 20 of 29 patients (69.0%) with open fractures, which leads to a divergence of the logistic regression algorithm. Patients with missing data on the fixation method (*n* = 22; 6.1%) were included in all analyses in which the corresponding variable was optional; Pairwise deletion was used for pairwise comparisons.

Given the limited number of events—17 cases of infection in the subgroup without osteomyelitis—and the low event-to-variable ratio (EPV ≈ 2.8), the results of the multivariate analysis were considered exploratory and interpreted with caution. Model quality was assessed using the Nagelkerke R^2^ and the Akaike Information Criterion (AIC).

The study was conducted in accordance with the principles of the Declaration of Helsinki. The study protocol was approved by the Local Bioethics Committee of the National Scientific Center of Traumatology and Orthopedics named after Academician N.D. Batpenov (Protocol No. 1/5 dated 6 March 2026). Due to the retrospective design of the study and the use of anonymized data, the need for individual informed consent was waived.

## 3. Results

### 3.1. Sample Characteristics and Patient Flow Dynamics

**Figure 1:**
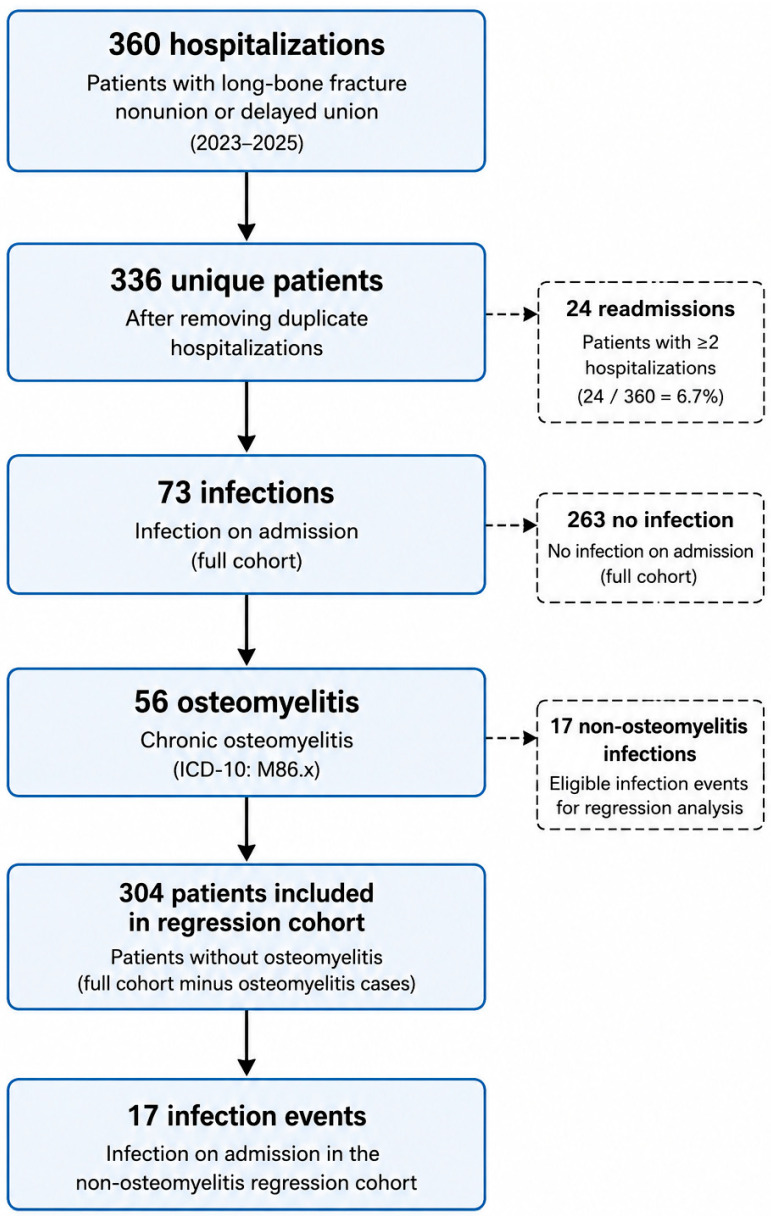
Study flow diagram showing hospitalizations, unique patients, infection cases, osteomyelitis cases, and the final cohort included in the multivariable regression analysis.

Over the three-year observation period (2023–2025), NSCTO recorded 360 hospitalizations in 336 unique patients with consolidation disorders; 24 patients were admitted twice (re-hospitalizations are discussed in detail in Section 3.6) (Figure 1).

**Figure 2:**
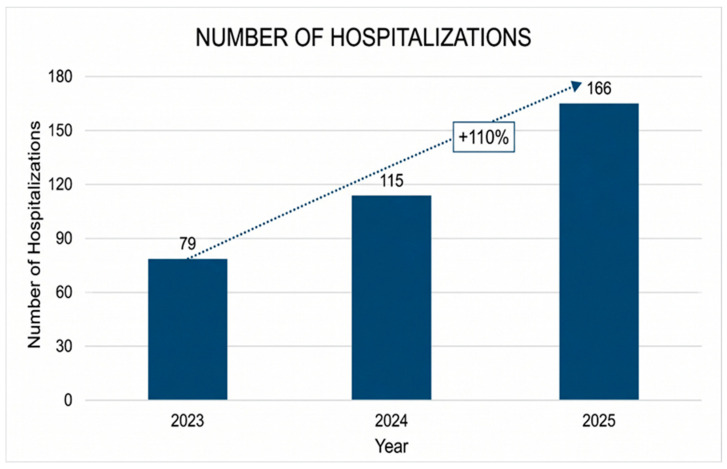
Annual trend in hospitalizations at NSCTO for fracture nonunion and delayed union during 2023–2025.

The annual patient flow increased consistently: 79 cases in 2023, 115 in 2024, and 166 in 2025, representing a more than twofold increase over the observation period (Figure 2). This observation indicates a growing number of admissions to the specialized center during the study period. However, the reasons for this increase cannot be determined from the present data and may reflect multiple factors, including changes in referral patterns, institutional capacity, case ascertainment, or coding practices.

**Figure 3:**
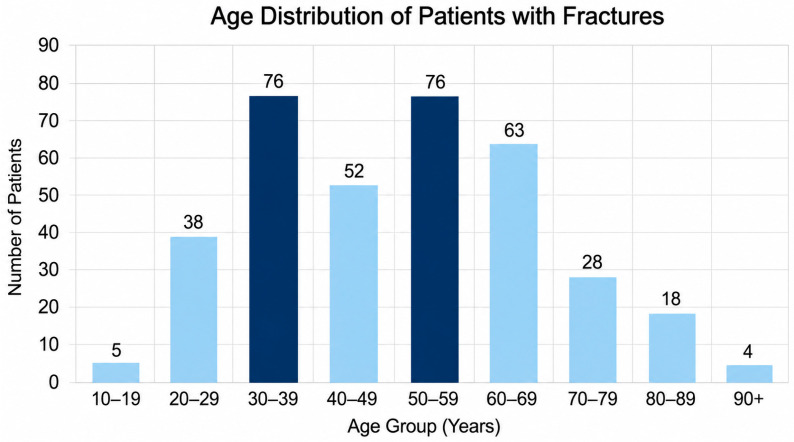
Distribution of patients by age groups (*n* = 360). The observed bimodal age distribution may reflect different injury patterns across age groups.

The cohort was predominantly female—189 patients (52.5%) versus 171 men (47.5%). The median age was 50 years (IQR 36–61; range 16–92). The distribution was bi-peak, with peaks in the 30–39 and 50–59 age groups (76 patients each) (Figure 3).

The average body mass index was 27.7 ± 5.8 kg/m^2^. Overweight or obesity (BMI ≥ 25 kg/m^2^) was recorded in 235 patients (65.3%), of whom 115 (31.9%) had obesity class I or higher. The baseline characteristics of the study cohort are summarized in Table 1.

### 3.2. Nosological Structure, Anatomical Distribution, and Timing of Presentation

The leading nosological entity was pseudoarthrosis (ICD-10: M84.1)—303 cases (84.2%). Delayed consolidation (M84.2) was diagnosed in 57 patients (15.8%).

The most frequently affected segment was the femur (130 cases; 36.1%), followed by the humerus (85 cases; 23.6%), and the tibia (54 cases; 15.0%). Together, these three segments accounted for almost three-quarters of the entire sample (74.7%), highlighting the disproportionately high load of nonunion on the supporting and functionally critical long bones. The predominant sites of injury were the middle third of the diaphysis (37.2%) and its proximal part (33.3%). Open fractures were recorded in 29 patients (8.1%); their relationship with the incidence of infection is discussed in Section 3.5.

The median interval from injury to hospitalization at NSCTO was 2 years (IQR 1–4). In total, 40.6% of patients were hospitalized within the first year after injury, while 17.1% were admitted more than five years later. These data indicate a high proportion of patients with long-standing impaired union and previous treatment prior to admission to a specialized center.

### 3.3. History of Previous Treatment: Burden of Failed Fixation

One of the most significant characteristics of this cohort was the high prevalence of previous surgical interventions. At the time of the primary fracture, the most common procedures used were locked intramedullary osteosynthesis (LIOS) (102 patients; 28.3%), plate osteosynthesis (92 patients; 25.6%), and external fixation apparatus (EFA) (38 patients; 10.6%). Other treatment methods were recorded in 35 patients (9.7%).

Notably, 93 patients (25.8%) had not received any surgical treatment, which may indicate both an initially conservative approach and limited access to surgical care at the regional level.

By the time of hospitalization at NSCTO, 123 patients (34.2%) had already undergone at least one attempt at revision osteosynthesis at other institutions. In other words, one in three patients presented not with primary fixation failure but with failed revision, representing the most complex and refractory end of the nonunion spectrum.

### 3.4. Surgical Tactics at NSCTO: Fixation and Biological Augmentation

At NSCTO, plate osteosynthesis was most frequently used (110 patients; 30.6%), followed by intramedullary nailing (IMN) (63 patients; 17.5%), external fixation device (61 patients; 16.9%), and total joint arthroplasty (52 patients; 14.4%). In 52 patients (14.4%), implant fixation was not used, primarily as part of staged procedures or debridement surgeries. In 22 cases (6.1%), complete data on the fixation method are unavailable due to incomplete primary documentation.

**Figure 4:**
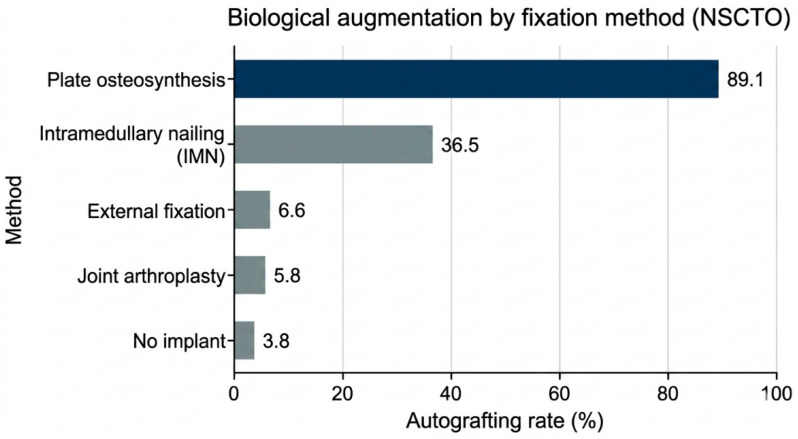
Frequency of autografting by surgical treatment method at NSCTO (*n* = 360).

A defining feature of the institutional protocol was the systematic combination of mechanical fixation and biological augmentation: bone autografting was performed in 181 patients (50.3%). The integration of autograft and plate osteosynthesis was particularly consistent: 98 of 110 patients in this group (89.1%) simultaneously underwent autografting, reflecting a targeted strategy of simultaneously addressing both mechanical and biological prerequisites for consolidation. When using external fixation devices (EFDs), autografting was used in only 4 of 61 cases (6.6%), indicating fundamentally different biological concepts depending on the chosen fixation method (Figure 4).

### 3.5. Infection, Hospital Stay, and Associated Factors of Severity and Resource Intensity of Treatment

Infection was recorded in 73 hospitalizations (20.3%) at admission. Of these, 56 hospitalizations had chronic osteomyelitis (M86.4/M86.6), while 17 patients had signs of infection associated with nonunion or delayed consolidation without M86.x coding. Infection was universal in osteomyelitis (56/56; 100%), while among patients without osteomyelitis, its frequency was 17/304 (5.6%).

The presence of infection was significantly associated with a longer hospital stay: the median hospital stay was 15 (IQR 10–22) in infected patients versus 9 (IQR 6–13) in uninfected patients (*p* < 0.001, Mann–Whitney test). Patients with osteomyelitis required a median of 17 hospital days (IQR 13–22) compared with 9 (IQR 7–14) for nonunion (*p* < 0.001). The results of the univariate analysis of factors associated with infection are presented in Table 2.

The most significant factors associated with infection were previous revision osteosynthesis (90.4% vs. 19.9%; *p* < 0.001), osteomyelitis (76.7% vs. 0%; *p* < 0.001), open fracture (27.4% vs. 3.1%; *p* < 0.001), and male sex (61.6% vs. 43.9%; *p* = 0.011). Diabetes mellitus, age, BMI, and the interval from injury to hospitalization did not demonstrate a statistically significant association with infection on admission.

To explore factors associated with infection on admission, a multivariable logistic regression analysis was performed. Patients with M86.x (*n* = 56) were excluded from the regression model due to the definitional dependence of the outcome. The final model included 304 hospitalizations without chronic osteomyelitis, of which 17 had infection on admission.

In the exploratory multivariable model, previous revision osteosynthesis prior to admission to NSCTO was the only factor associated with infection on admission (OR 8.26; 95% CI 2.76–24.74; *p* < 0.001). Other variables—diabetes mellitus, age, sex, BMI, and the interval from injury to hospitalization at NSCTO—did not demonstrate statistically significant associations with infection (Table 3). The model had an acceptable quality of fit (Nagelkerke R^2^ = 0.152; AIC = 128.3). However, due to the limited number of events (EPV ≈ 2.8), the results should be interpreted with caution and considered exploratory.

**Figure 5:**
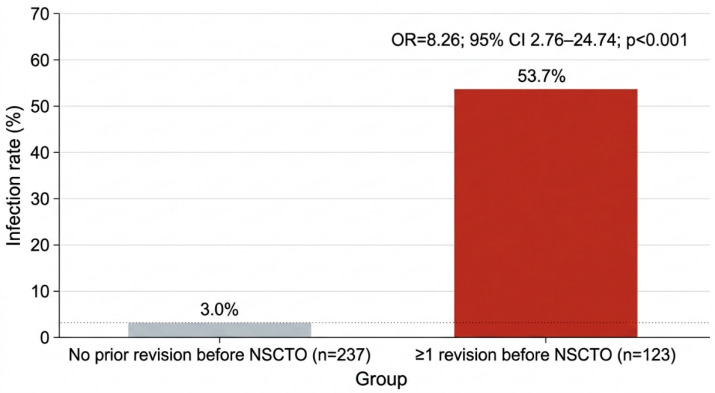
Infection rate on admission by the presence of prior revision osteosynthesis (*n* = 360).

The association between prior revision osteosynthesis and infection was particularly strong. Among patients with infection, 90.4% had undergone previous revision osteosynthesis compared with 19.9% of patients without infection (*p* < 0.001). In the multivariable analysis, previous revision osteosynthesis was associated with infection (OR 8.26; 95% CI 2.76–24.74; *p* < 0.001), supporting its role as a marker of clinical complexity and prior treatment burden. The incidence of infection according to previous revision osteosynthesis status is shown in Figure 5.

### 3.6. Readmissions: Patterns of Relapse and Treatment Failure

Of the 336 unique patients, 24 (7.1%) required readmission to NSCTO during the observation period. All readmissions were limited to two episodes. Of these, 15 (62.5%) were readmitted in a different calendar year, ruling out planned staging and indicating true relapse or incomplete resolution of the underlying pathology.

Classification by anatomical and diagnostic features revealed that 16 patients (66.7%) returned with the same diagnosis in the same bone segment—the most operational definition of treatment failure. Four patients (16.7%) had a different diagnosis in the same segment upon readmission, most commonly progression from nonunion to osteomyelitis. Another four patients (16.7%) were readmitted for an independent pathology involving a different anatomical location. Among patients who experienced readmission, 45.8% had undergone previous revision osteosynthesis compared with 35.9% among patients with a single hospitalization (*p* = 0.096), although this difference did not reach statistical significance.

The dynamics of infection between hospitalizations were clinically informative: four patients had active infections during both admissions, forming a subgroup with refractory chronic osteomyelitis. In one patient, the infection developed between hospitalizations, and in another, it resolved, reflecting the heterogeneous and unpredictable course of fracture-related infection in this population.

This analysis is limited to the inpatient follow-up period; long-term outcome data were not assessed (see Section 4.6 for more details).

## 4. Discussion

### 4.1. Relevance of the Problem and Burden of Nonunion

Fracture nonunion is one of the most clinically and economically significant complications in modern traumatology. According to a large population-based study by Zura et al., which included more than 4 million patients, nonunion occurs in approximately 4.9% of extremity fractures; the risk varies significantly depending on the location, severity of injury, and comorbidity of the patients [2].

A systematic review and meta-analysis by Tian et al., including 111 studies and 15 potential predictors, reported that infection, open fracture, age over 60 years, and high-energy mechanism of injury were consistently associated with an increased risk of tibial nonunion [5,6].

The observed increase in hospitalizations during the study period should be interpreted cautiously. Although it may reflect greater utilization of specialized care for patients with impaired fracture healing, alternative explanations such as changes in referral pathways, institutional capacity, healthcare organization, or coding practices cannot be excluded. The present study was not designed to determine the causes of this trend.

The functional consequences of nonunion are severe and long-term. Tay et al., in a cohort study of 423 patients with femoral and tibial shaft fractures, showed that the physical component of the SF-12 did not improve over a 12-month follow-up period in patients with impaired union, while improvements were observed in the normal union group. Moreover, even among patients with normal union, only 72% returned to work after one year, and 54% continued to experience pain—the results in the nonunion group were significantly worse for both measures [7]. Such findings emphasize the substantial clinical and socioeconomic burden of impaired fracture healing and highlight the importance of timely recognition and appropriate management of patients with nonunion and delayed union.

### 4.2. The Role of a Specialized Center and the Consequences of Late Referral

The median interval between the initial injury and referral to a specialized center was 2 years, and 17.1% of patients presented more than five years after injury. These findings suggest that many patients were referred only after prolonged periods of impaired fracture healing and multiple previous treatment attempts. Previous studies have reported worse functional outcomes among patients with prolonged nonunion and delayed healing; however, the present study was not designed to evaluate the effect of referral timing on clinical outcomes [7].

From a pathophysiological perspective, prolonged nonunion and repeated surgical interventions may theoretically contribute to the deterioration of both the biological and mechanical conditions of healing, consistent with the principles of the diamond concept [8].

In our cohort, this is confirmed by the high proportion of patients (34.2%) referred after failed revision surgeries, reflecting the most complex and refractory patient population.

### 4.3. Association Between Previous Revision Osteosynthesis and Infection at Admission

The key finding of this study is the strong association between previous revision osteosynthesis and the presence of infection at admission. In the exploratory multivariable model, previous revision osteosynthesis was associated with infection; however, this finding should be interpreted cautiously.

Given the retrospective design of the study and the limited number of events (EPV ≈ 2.8), the association may reflect both a true biological effect and the influence of residual confounding. In particular, revision surgery is likely a marker of more severe initial injury, compromised soft tissue, and failure of previous treatment.

Furthermore, because all variables were assessed at the time of admission, the temporal relationship between revision surgery and infection could not be established. Therefore, it remains unclear whether infection developed after revision surgery, whether infection contributed to treatment failure and the need for revision, or whether both conditions reflect underlying injury severity and treatment complexity.

From a biological perspective, repeat surgical interventions may contribute to progressive periosteal devascularization, hematoma formation, and an increased risk of bacterial colonization, which is consistent with current understanding of the pathogenesis of implant-associated infection [9,10]. However, the present study was not designed to determine whether such mechanisms were responsible for the observed association.

Thus, previous revision osteosynthesis should be considered primarily as a marker of a more complex clinical course rather than a proven causal factor for infection. The observed association may reflect the cumulative burden of severe injury, repeated interventions, impaired biology, and previous treatment failure. A causal relationship could not be definitively established within the framework of this study.

It is noteworthy that male gender, which showed a significant association with infection in the univariate analysis (61.6% vs. 43.9%; *p* = 0.011), lost this association in the multivariate model (OR 1.53; *p* = 0.454), likely due to unmeasured injury-related factors and the limited statistical power of the model [11].

### 4.4. Lack of a Single Standard of Treatment and Current Therapeutic Concepts

Over the past decades, the lack of a single standard for the treatment of long-bone nonunions has become firmly established in the literature, owing to the heterogeneity of the etiology, biological conditions, and mechanical defects in each individual case. The “diamond concept” formulated by Andrzejowski and Giannoudis has received the most widespread recognition as a conceptual basis for treatment planning. It postulates that successful consolidation requires the simultaneous achievement of four conditions: mechanical stability, osteogenic cells, osteoinductive signals, and an osteoconductive scaffold. Application of all four principles ensures a combined consolidation rate of approximately 90% [8].

Bone autografting remains the most widely used method of biological augmentation for nonunions—in our cohort, it was performed in 50.3% of patients and in 89.1% of those who underwent plate osteosynthesis. Sen and Miclau, in a review in the journal Injury, emphasize that iliac crest autograft simultaneously provides osteoconduction, osteoinduction, and osteogenesis, which determines its central role in the treatment of nonunions [12]. However, donor site complications during harvesting from the iliac crest are significant; RIA (Reamer-Irrigator-Aspirator) systems for harvesting intramedullary autograft with lower donor morbidity are being actively studied as an alternative, as well as bone tissue substitutes—allograft, synthetic scaffolds, growth factors (BMP-2, BMP-7), and bone marrow aspirate concentrate (BMAC) [13]. The lack of randomized controlled trials with sufficient power for most biological adjuvants does not allow for the formulation of unambiguous recommendations, which determines the thesis about the absence of a single standard of treatment in both the domestic and international literature [5,6].

### 4.5. Study Limitations

This study has several important limitations that should be considered when interpreting the findings. The retrospective single-center design limits external validity and does not allow causal relationships to be established. Because all variables were assessed at the time of admission, the temporal relationship between previous revision surgery and infection could not be determined.

The multivariable analysis was performed after exclusion of patients with chronic osteomyelitis because infection represents an integral component of this diagnosis. Consequently, the regression model applies primarily to patients with nonunion or delayed union without established chronic osteomyelitis and should not be generalized to the entire population of patients with impaired fracture healing.

Interpretation of the regression results is further limited by the small number of infection events in the non-osteomyelitis subgroup (17 events among 304 hospitalizations; EPV ≈ 2.8), which increases the risk of overfitting and unstable effect estimates. Therefore, the identified associations should be considered exploratory and hypothesis-generating rather than confirmatory. In addition, open fractures could not be retained in the final multivariable model because of sparse data and model instability, leaving the possibility of residual confounding related to injury severity.

Several clinically relevant variables were unavailable or incompletely documented in the retrospective records, including smoking status, injury mechanism, Gustilo–Anderson classification, number of previous operations, implant status, microbiological findings, inflammatory markers, vascular comorbidities, nutritional status, renal disease, steroid use, and glycemic control. The absence of these variables may have resulted in residual confounding and limited the ability to fully adjust for infection risk. Furthermore, the exact date of injury was not consistently available in all records, preventing reliable reporting of referral delays in months.

The unit of analysis was hospitalization rather than the individual patient. Although repeat admissions represented only 24 of 360 hospitalizations (6.7%), the inclusion of multiple admissions from the same patient may have introduced within-patient correlation that was not formally accounted for in the statistical analysis.

Long-term outcomes, such as fracture union, infection eradication, functional recovery, reoperation rates, return to work, and patient-reported quality of life, were not systematically available because structured follow-up data were not collected. Finally, as a national tertiary referral center, NSCTO predominantly manages severe and treatment-resistant cases, which may limit the generalizability of the findings to broader orthopedic populations.

### 4.6. Clinical and Practical Significance

These findings have important practical implications. The high proportion of patients presenting after previous unsuccessful procedures and prolonged periods of impaired fracture healing underscores the complexity of cases managed at specialized referral centers [7]. From a surgical perspective, the frequent combination of stable fixation and biological augmentation is consistent with the principles of the diamond concept [8] and reflects the widespread use of a combined mechanical–biological approach to nonunion management in contemporary orthopedic practice [12,13].

## 5. Conclusions

Patients with nonunion and delayed union of long-bone fractures referred to a specialized orthopedic center represent a clinically complex population characterized by prolonged time from injury, a high prevalence of previous surgical interventions, and a substantial burden of infection. In an exploratory multivariable analysis, previous revision osteosynthesis was associated with infection on admission and may represent a marker of clinical complexity and prior treatment burden rather than a causal determinant of infection.

These findings provide insight into the characteristics of patients managed at a national tertiary referral center and highlight the challenges associated with advanced and treatment-resistant cases of impaired fracture healing. Given the retrospective design, selective referral population, and limited number of infection events included in the regression model, the observed associations should be interpreted with caution. Further prospective studies are needed to evaluate treatment outcomes and to clarify factors associated with infection and successful fracture healing in this patient population.

## Figures and Tables

**Table 1:** Baseline characteristics of the study cohort (*n* = 360).

Variable	Value
Hospitalizations	360
Unique patients	336
Readmissions	24 (6.7%)
Age, years, median (IQR)	50 (36–61)
Male sex	171 (47.5%)
Female sex	189 (52.5%)
BMI, kg/m^2^, mean ± SD	27.7 ± 5.8
BMI ≥ 25 kg/m^2^	235 (65.3%)
Obesity (BMI ≥ 30 kg/m^2^)	115 (31.9%)
Nonunion (M84.1)	303 (84.2%)
Delayed union (M84.2)	57 (15.8%)
Femur	130 (36.1%)
Humerus	85 (23.6%)
Tibia	54 (15.0%)
Other locations	91 (25.3%)
Open fracture	29 (8.1%)
Previous revision osteosynthesis	123 (34.2%)
Infection on admission	73 (20.3%)
Osteomyelitis (M86.x)	56 (15.6%)
Time from injury to hospitalization, years, median (IQR)	2 (1–4)

Abbreviations: BMI, body mass index; IQR, interquartile range; SD, standard deviation. Note: Data are presented as *n* (%) unless otherwise indicated.

**Table 2:** Comparison of hospitalizations with and without infection on admission.

Variable	Infection (*n* = 73)	No Infection (*n* = 287)	*p*-Value
Previous revision osteosynthesis	66 (90.4%)	57 (19.9%)	<0.001
Open fracture	20 (27.4%)	9 (3.1%)	<0.001
Osteomyelitis (M86.x)	56 (76.7%)	0 (0%)	<0.001
Male sex	45 (61.6%)	126 (43.9%)	0.011
Diabetes mellitus	4 (5.5%)	16 (5.6%)	1.000
Age, years, median (IQR)	47 (34–60)	51 (37–62)	0.098
BMI, kg/m^2^, median (IQR)	27.4 (24.0–31.8)	27.0 (23.5–30.8)	0.619
Time from injury to hospitalization, years, median (IQR)	2.0 (1.0–5.5)	2.0 (1.0–3.0)	0.180

Abbreviations: BMI, body mass index; IQR, interquartile range. Note: Data are presented as *n* (%) unless otherwise indicated.

**Table 3:** Multivariate logistic regression analysis of factors associated with infection (non-osteomyelitis subgroup, *n* = 304).

Variable	OR	95% CI	*p*-Value
Previous revision osteosynthesis before admission to NSCTO	8.26	2.76–24.74	<0.001
Diabetes mellitus	0.92	0.09–8.99	0.944
Age (years)	1.01	0.97–1.05	0.714
Male sex	1.53	0.50–4.69	0.454
BMI (kg/m^2^)	1.02	0.93–1.12	0.663
Time from injury to hospitalization at NSCTO	0.94	0.81–1.09	0.410

Abbreviations: BMI, body mass index; CI, confidence interval; NSCTO, National Scientific Center of Traumatology and Orthopedics named after Academician N.D. Batpenov; OR, odds ratio. Note: Osteomyelitis (M86.x) was excluded from the multivariable model because of definitional collinearity with infection at admission. Due to the low events-per-variable ratio (EPV ≈ 2.8), the regression model should be considered exploratory and interpreted with caution.

## Data Availability

The data presented in this study are available from the corresponding author upon reasonable request. The data are not publicly available due to institutional and patient confidentiality restrictions.
